# Relationship between Vitamin C Deficiency and Cognitive Impairment in Older Hospitalised Patients: A Cross-Sectional Study

**DOI:** 10.3390/antiox11030463

**Published:** 2022-02-26

**Authors:** Yogesh Sharma, Alexandra Popescu, Chris Horwood, Paul Hakendorf, Campbell Thompson

**Affiliations:** 1 College of Medicine & Public Health, Flinders University, Adelaide 5042, Australia; 2 Department of General Medicine, Division of Medicine, Cardiac & Critical Care, Flinders Medical Centre, Adelaide 5042, Australia; 3 Department of Geriatrics & Rehabilitation, Flinders Medical Centre, Adelaide 5042, Australia; alexandra.popescu@sa.gov.au; 4 Department of Clinical Epidemiology, Flinders Medical Centre, Adelaide 5042, Australia; chris.horwood@sa.gov.au (C.H.); paul.hakendorf@sa.gov.au (P.H.); 5 Discipline of Medicine, The University of Adelaide, Adelaide 5005, Australia; campbell.thompson@adelaide.edu.au

**Keywords:** vitamin C deficiency, cognitive impairment, geriatric patients, older hospitalised patients, clock drawing test, mini mental state examination

## Abstract

Vitamin C is a powerful antioxidant and facilitates neurotransmission. This study explored association between vitamin C deficiency and cognitive impairment in older hospitalised patients. This prospective study recruited 160 patients ≥ 75 years admitted under a Geriatric Unit in Australia. Cognitive assessment was performed by use of the Mini-Mental-State-Examination (MMSE) and patients with MMSE scores <24 were classified as cognitively-impaired. Fasting plasma vitamin C levels were determined using high-performance-liquid-chromatography. Patients were classified as vitamin C deficient if their levels were below 11 micromol/L. Logistic regression analysis was used to determine whether vitamin C deficiency was associated with cognitive impairment after adjustment for various covariates. The mean (SD) age was 84.4 (6.4) years and 60% were females. A total of 91 (56.9%) were found to have cognitive impairment, while 42 (26.3%) were found to be vitamin C deficient. The mean (SD) MMSE scores were significantly lower among patients who were vitamin C deficient (24.9 (3.3) vs. 23.6 (3.4), *p*-value = 0.03). Logistic regression analysis suggested that vitamin C deficiency was 2.9-fold more likely to be associated with cognitive impairment after adjustment for covariates (aOR 2.93, 95% CI 1.05–8.19, *p*-value = 0.031). Vitamin C deficiency is common and is associated with cognitive impairment in older hospitalised patients.

## 1. Introduction

Vitamin C, also referred to as ascorbic acid, is a powerful antioxidant that cannot be synthesised by humans and some other primates due to the lack of an enzyme called gulonolactone oxidase [1]. Vitamin C plays an essential biological role by acting as a co-factor for a number of enzymes, which are required for proper functioning across a number of organs and tissue systems [2]. In the gastrointestinal tract, vitamin C helps in the absorption of non-heme iron and is involved in the formation of bile acids via cholesterol hydroxylation [2,3]. In addition, it plays a role in immune function and is involved in the synthesis of corticosteroids, aldosterone, adrenal hormones and in the formation of collagen [4,5]. Severe vitamin C deficiency results in development of scurvy, which can manifest as bleeding, fatigue, bony pains, skin manifestations such as perifollicular haemorrhages, petechiae and ecchymosis [6].

Vitamin C plays a significant role in the functioning of the brain by regulating neurotransmitter synthesis and release [7]. These functions include acting as a co-factor for dopamine beta-hydroxylase, which converts dopamine to noradrenaline. Vitamin C is involved in the modulation of glutamatergic, dopaminergic, cholinergic and GABAergic neurotransmission and regulates the release of catecholamines and acetylcholine from synaptic vesicles [8,9]. In addition, the antioxidant properties of vitamin C limit damage caused by ischaemia-reperfusion mediated injury and protects against glutamate excitotoxicity [9,10].

Previous studies [11,12,13] indicate that vitamin C deficiency may have a role in neurocognitive dysfunction and may be associated with cognitive impairment, depression and confusion. Two cross-sectional studies [11,14] have linked lower vitamin C status with greater cognitive impairment. However, an Australian study [12] in otherwise healthy volunteers found no association between cognitive dysfunction and vitamin C deficiency. A systematic review by Travica et al. [7], which included 50 studies, found no correlation between vitamin C levels and cognition, however, the majority of studies included in this review involved community-dwelling healthy participants with a higher baseline cognitive performance, which could have narrowed the chance of detecting cognitive effects of vitamin C. In addition, the studies included in this meta-analysis had limitations in terms of handling of blood samples and biochemical analyses because underestimation of vitamin C concentrations could occur if the sample was not protected from light and not transported on ice [7,15].

Furthermore, some of the studies till date have other methodological limitations namely: use of small sample size and a variable definition for classification of patients as vitamin C deficient, with one study [12] classifying patients with vitamin C levels below 28 μmol/L as deficient while another study using even higher cut-off levels [16]. Evidence suggests that clinical manifestations of scurvy usually develop once vitamin C levels drop below 11.4 μmol/L [17,18], thus it is possible that cognitive dysfunction may not be present at a higher vitamin C level and manifests only in patients who have a severe vitamin C deficiency. The present study investigated the relationship between cognitive status and vitamin C deficiency in older hospitalised patients unit using lower vitamin C cut-off levels, which usually result in clinical manifestations of scurvy. The hypothesis for this research was that vitamin C deficiency is common in older hospitalised patients and that patients with severe vitamin C deficiency will have lower cognitive scores.

## 2. Materials and Methods

Patients ≥ 75 years who were admitted to a geriatric unit of Flinders Medical Centre, Adelaide, South Australia, were recruited by convenience sampling in this research. Written informed consent was obtained from the participants, and, in cases of cognitive impairment, consent was obtained from the legal guardian. The exclusion criteria were lack of valid consent, patients receiving end-of-life care, and those on vitamin C replacement. Ethical approval for this study was granted by the Southern Adelaide Human Clinical Research Ethics Committee (approval no 64.190, dated 9 August 2019) and this study was registered with Australia and New Zealand Clinical Trial Registry.

### 2.1. Patient and Public Involvement

Cognitive impairment is highly prevalent in older hospitalised patients and, therefore, the study results are likely to be high priority for patients. However, patients were not directly involved in the study design, conduct or outcomes of this research project.

Cognitive status was determined by use of the Mini Mental State Examination (MMSE) [19] and the Clock-Drawing Test (CDT). The MMSE is scored on a 30-point scale and uses items that assess: orientation (temporal and spatial, 10 points), memory (registration and recall, 6 points), attention and concentration, 5 points, language (verbal and written, 8 points), and visuospatial function (1 point) [19]. While different cut-off points were used across different studies [20], for this study MMSE scores below 24 were regarded as abnormal and indicative of cognitive impairment. While the MMSE was originally developed to identify cognitive impairment among psychiatric patients [21], it was subsequently validated for use as a screening tool for dementia across a wide range of patients in both outpatient and inpatient settings [22,23].

We used the CDT in addition to the MMSE because studies indicate that the CDT is highly sensitive and specific in the detection of mild dementia and is reasonably accurate in separating patients with mild cognitive impairment (MCI) from healthy patients, and the combination of the CDT with the MMSE enhances the psychometric properties of these scales and is valid for detection of dementia [24,25]. The CDT was performed by providing the participants with a 10 cm pre-drawn circle on a piece of paper, and they were asked to draw an analogue clock, including all the numbers, and set the clock hands to a specified time of 10 past 1100 h. Performance on the CDT depends upon a combination of visuospatial ability, executive function, motor function, attention, numerical knowledge and language comprehension [26]. Patients were scored on a simple subjective qualitative interpretation of clock drawing as normal (without error) and abnormal (with error) as suggested by Sleutjes et al. [23].

Mood can affect cognition and was assessed by using the Geriatric Depression Scale (GDS) [27]. GDS is a 15-item tool that has been validated for screening depressive symptoms in the older population including acutely hospitalised medical patients [28,29]. Frailty also relates strongly to impaired cognition. Its assessment was performed by use of the Edmonton Frail Scale (EFS). The EFS is a valid and reliable instrument for identification of frailty in hospitalised patients and predicts clinical outcomes [30,31]. The EFS contains 9 components and is scored out of 17. Individual components include cognition, general health status, self-reported health, functional independence, social support, polypharmacy, mood, continence and functional performance. The component scores are summed, and the following cut-off scores are used to classify the severity of frailty: not frail (0–5), apparently vulnerable (6–7), mild frailty (8–9), moderate frailty (10–11) and severe frailty (12–17). For this study, patients with EFS scores ≥8 were classified as frail and those with EFS scores <8 as non-frail.

Nutritional risk was determined by the use of the Malnutrition Universal Screening Tool (MUST) [32]. Fruit and vegetable consumption was determined by asking the patients their approximate daily intake of standard portions/day in the week prior to their admission to the hospital. Patients were specifically examined for any signs suggestive of scurvy, namely: ecchymosis, bruising, gingivitis and perifollicular hyperkeratosis [33]. Impairment of mobility and gait were risk factors for dementia thus the activities of daily living (ADL) were assessed by use of the Hospital Admission Risk Profile (HARP) score [34], which predicts patients at high risk of discharge to a facility. We determined the sociodemographic status of the participants by including the following variables: living status (whether living alone or with a partner), education level (secondary school or a higher university degree) and annual income (≥ or <AUD 40,000/year). Polypharmacy was defined as being on 5 or more medications. Medications with anticholinergic activity, which can impact cognition (such as the use of antihistamines, antiparkinson, opiates, antimuscarinic, antipsychotic and antiepileptic drugs) [35] were also determined.

A trained phlebotomist obtained fasting blood samples to determine vitamin C levels. The blood sample was wrapped in an aluminium foil and immediately placed on ice for transport to a central laboratory. High performance liquid chromatography (HPLC) was used to determine vitamin C levels. HPLC has been previously validated for rapid and specific measurement of vitamin C [36]. Plasma vitamin C levels correlate with dietary vitamin C intake, and unlike leucocyte vitamin C levels, plasma vitamin C levels are not influenced by changes in the white blood cell (WBC) count and thus represent an accurate measure of vitamin C status [36,37]. According to Johnston’s criteria [38], vitamin C levels ≥28 μmol/L are classified as normal, 11–27 μmol/L as vitamin C depletion, and <11 μmol/L as vitamin C deficiency. In addition, blood samples were drawn for the determination of haemoglobin, creatinine, C-reactive protein (CRP), vitamin D and vitamin B12 levels. The haemoglobin and creatinine levels were determined using spectrophotometry, while C-RP, vitamin D and vitamin B12 levels were determined by a rapid immunoassay, Roche Diagnostics (https://www.roche-australia.com) (accessed on 1 May 2020), in a central laboratory.

### 2.2. Statistics

The normality of the data was assessed by visual inspection of the histograms. Continuous variables were assessed by use of the student *t* tests or rank-sum tests and categorical variables by Chi-squared statistics or Fisher’s exact test as appropriate. Patients with MMSE scores ≥24 were classified as having normal cognition, while those with MMSE scores <24 as cognitively impaired. For this study, patients with vitamin C levels <11 μmol/L were defined as vitamin C deficient and were compared with the group whose vitamin C levels were ≥11 μmol/L.

Logistic regression analysis was used to determine whether vitamin C deficiency was associated with cognitive impairment after adjustment for the following covariates: age, sex, Charlson index, MUST score, HARP score, depression, living status (whether alone), education level, socioeconomic status, fruit/vegetable intake, polypharmacy, haemoglobin, creatinine, vitamin D and vitamin B12 levels.

The use of logistic regression model with the use of small to moderate sample sizes may sometimes lead to an introduction of an analytical bias, which may result in overestimation of the effect size. Corrective measures were applied by the performance of sensitivity analysis with the use of the bootstrap method as suggested by Nemes et al. [39] and bootstrapped standard errors (SE) with 95% confidence intervals were generated. In addition, a prediction graph with 95% confidence intervals was plotted to determine the probability of vitamin C deficiency at different MMSE scores using the margins plot command in STATA.

The sample size for this study was based on a pilot study involving 20 older hospitalised patients, which found that the mean (SD) MMSE scores were 27 (7.5) in patients who were vitamin C replete compared to 22 (12.5) in vitamin C deficient patients, with an alpha level of 0.05 and power of 80% the calculated sample size was 136 and assuming 15% missing data 156 patients were thought to be sufficient for this study. All statistical analyses were conducted using Stata version 17.0 (StataCorp, College Station, TX, USA).

## 3. Results

**Figure 1:**
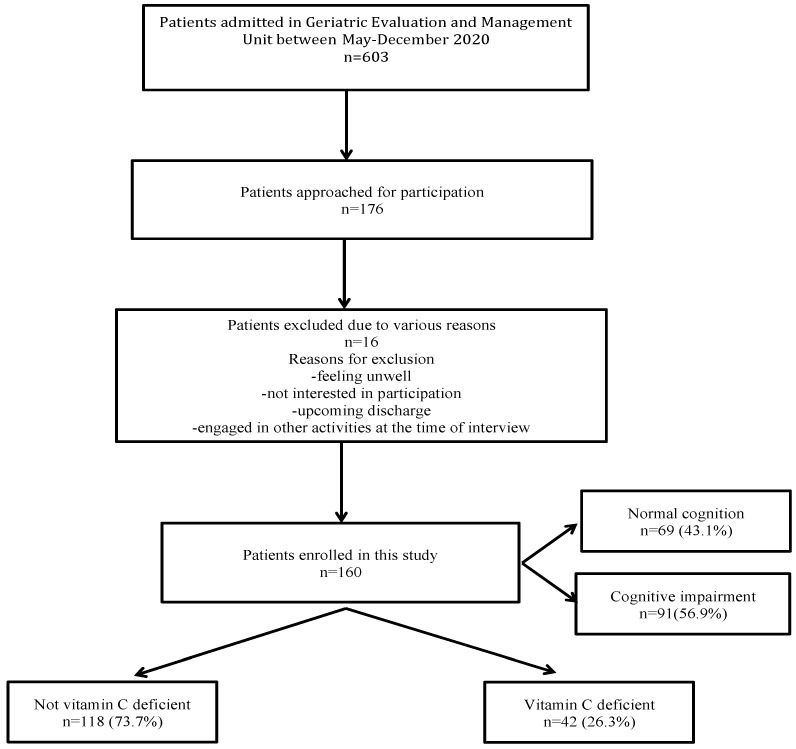
Study flow diagram.

A total of 603 patients were admitted under the geriatric unit between May-December 2020, of whom, 176 patients were approached by convenient sampling for participation and 160 patients were recruited for this study (Figure 1). The characteristics of patients who were not approached for participation were not significantly different from those who were included in this study in terms of age, sex, Charlson index, living status and length of hospital stay (LOS) (*p* > 0.05). The mean (SD) age was 84.4 (6.4) years range (73–105 years) and 96 (60%) were females. All patients were residing in their own homes and 78 (48.7%) were living with their partners. The mean Charlson index was 8.4 (2.6) and the majority of patients were on polypharmacy (130; 81.3%) and many were admitted with falls as the principal diagnosis (69, 43.1%). The mean (SD) MMSE score was 24.6 (3.4) (range 19–30). A total of 69 (43.1%) patients had normal cognition (MMSE score ≥ 24) and 91 (56.9%) were found to have cognitive impairment (MMSE score < 24). Patients with cognitive impairment were older, with a higher Charlson index and frailty scores and were less likely to have a university degree than cognitively intact patients (*p* < 0.05). However, there was no difference with regards to gender, nutrition status, marital status and number of medications between the cognitively normal and impaired groups (*p* > 0.05). The mean (SD) vitamin C levels were 26.8 (23.0) μmol/L, (range 3–148). The median (IQR) time from hospital admission to the collection of vitamin C sample was 4 (4, 4) days. A total of 118 (73.7%) patients were not vitamin C deficient (vitamin C levels ≥ 11 μmol/L), while 42 (26.3%) were classified as vitamin C deficient (levels <11 μmol/L) (Figure 1).

The median (IQR) time for the collection of the vitamin C sample after hospital admission was not significantly different between patients who were not vitamin C deficient compared to those who had vitamin C deficiency (4 (4, 3) vs. 4 (4, 4) days, *p*-value = 0.095). Patients with vitamin C deficiency were more likely to be current smokers with a higher Charlson index and mean creatinine level than patients who were not vitamin C deficient (Table 1). When compared to patients who were not vitamin C deficient, the mean (SD) MMSE scores were significantly lower among patients who were vitamin C deficient (24.9 (3.3) vs. 23.6 (3.4), *p*-value = 0.03). However, there was no difference in the proportion of patients who made errors on the CDT between the two groups (Table 1).

**Figure 2:**
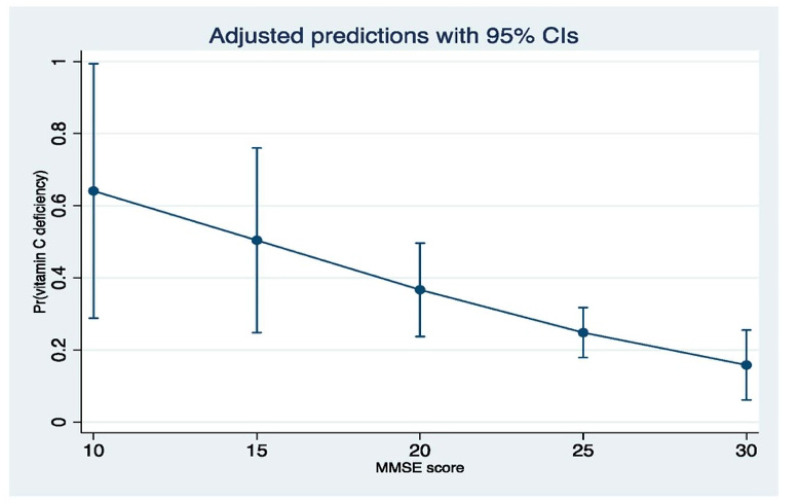
Prediction probability of vitamin C deficiency according to MMSE scores.

Logistic regression analysis suggested that vitamin C deficiency was 2.9-fold more likely to be associated with cognitive impairment after adjustment for age, sex, Charlson index, MUST score, HARP score, depression, living status (whether alone), education level, socioeconomic status, fruit/vegetable intake, polypharmacy, haemoglobin, creatinine, vitamin D and vitamin B12 levels (aOR 2.93, 95% CI 1.05–8.19, *p*-value = 0.031) (Table 2). Sensitivity analysis confirmed that vitamin C deficiency was associated with cognitive impairment after adjustment for the above-mentioned covariates (Coefficient 1.03, Bootstrap SE 0.50, 95% CI 0.05–2.03, *p*-value 0.039). The margins plot suggested that lower MMSE scores increases the probability of being diagnosed with vitamin C deficiency (Figure 2).

## 4. Discussion

A substantial proportion (26.3%) of older hospitalised patients were vitamin C deficient. Only a few clinical characteristics, namely a history of current smoking and higher Charlson index and creatinine levels predicted vitamin C deficiency. Vitamin C deficiency was associated with an increased risk of cognitive impairment as assessed by the MMSE scores but not when assessed by the CDT even after adjustment for a number of covariates.

The results of our study corroborate previous evidence [3,40,41] that a high proportion of older hospitalised patients have vitamin C deficiency. Interestingly, our study indicates that there are only a few clinical correlates, which can predict a low vitamin C of the home-dwelling but currently hospitalised elderly. Furthermore, according to this study, the symptoms, which were compatible with the diagnosis of scurvy, were not significantly different among patients with or without vitamin C deficiency. Scurvy is characterised by prominent skin manifestations, including perifollicular hyperkeratosis, cork-screw hairs, gingival bleeding, petechiae and ecchymosis [17,42] Bruising and bleeding, which characterise scurvy, can be seen in older hospitalised patients because of a number of reasons such as falls [43], senile purpura (which occurs because of increased skin fragility associated with ageing) [44] and the adverse effects of commonly administered medications such as antiplatelet agents, anti-coagulants and glucocorticoids [45]. Moreover, perifollicular hyperkeratosis, which is regarded as a hallmark of scurvy, may be difficult to differentiate from leukocytoclastic vasculitis [46]. It may, therefore, be difficult to diagnose vitamin C deficiency solely on clinical grounds in older hospitalised patients. Given the high prevalence of vitamin C deficiency in hospitalised patients, there is a need for heightened vigilance, and biochemical confirmation of vitamin C status is required in suspected cases.

This study indicates that vitamin C deficiency was associated with cognitive impairment as reflected by lower MMSE scores in vitamin C deficient patients when compared to those who were not vitamin C deficient. This association remained significant after adjustment for not only age but also various factors, which can be associated with cognitive impairment such as a higher number of comorbidities as determined by the Charlson index, education level, depression, socioeconomic status, polypharmacy, haemoglobin, creatinine, vitamin D and B12 levels [47,48,49,50,51,52]. Our study results are in line with a study by Gale et al. from the UK [14], which involved 921 community-dwelling older people ≥ 65 years. Their study found that patients with moderate to severe vitamin C deficiency were 1.6 fold (OR 1.6, 95% CI 1.1–2.3) more likely to be diagnosed with cognitive impairment assessed by use of the Hodgkinson abbreviated mental test [53]. Similarly, another recent study [11] from New Zealand, which included a cohort of 404 people aged 49–51 years, found that the odds of mild cognitive impairment, as determined by Montreal Cognitive Assessment (MOCA) [54], were twice as high for individuals whose vitamin C levels were below 23 μmol/L (OR 2.1, 95% CI 1.2–3.7, *p* = 0.01). However, our study results are contrary to an Australian study, which included healthy adults aged 24–96 years and assessed cognitive function by use of a battery of cognitive screening tools: the Modified Mini Mental State Examination (3MS) [55], the Revised Hopkins Verbal Learning Test (HVLT-R) [56], the Symbol Digits Modalities Test (SDMIT) [57] and the Swinburne University Computerised Cognitive Assessment Battery (SUCCAB) [12]. That study found that there was no difference with respect to the diagnosis of major cognitive impairment with 3MS test among patients with adequate or inadequate vitamin C status. However, patients who were in the adequate vitamin C group had significantly higher scores on measures of recognition, immediate and delayed recall assessed by the HVLT-R and on SDMT screening when compared to vitamin C inadequate group. Finally, using the SUCCAB, that study found that, although the accuracy to reaction time was significantly higher in the adequate vitamin C group for certain tasks, there was no difference with respect to measures of episodic memory or general alertness and motor speed when compared to the vitamin C inadequate group. The discrepancy in the results of this study compared to our study, in terms of cognition, could be related to their inclusion of much younger patients and likely healthier patients with a wide age range from the community compared to older hospitalised patients in our study. In addition, that study used higher vitamin C cut off levels (<28 μmol/L vs. <11 μmol/L) for diagnosing vitamin C deficiency compared to our study. It is possible that cognitive dysfunction is apparent only with severely low vitamin C status (i.e., vitamin C levels < 11 μmol/L) and may not manifest with less severe degrees of hypovitaminosis C (11–28 μmol/L). In support of this conjecture, a re-analysis of our own data using these authors’ looser definition of vitamin C deficiency showed no significant alteration of cognition in the deficient subjects (data not shown). Subtle changes in cognition might be detectable with a less severe deficiency of vitamin C [12]. Animal studies [58,59] have indicated that higher supplementation of vitamin C reduced amyloid plaque burden in the cortex and hippocampus in mice with resultant amelioration of blood–brain barrier disruption and mitochondrial alteration. However, evidence in relation to the benefits of vitamin C supplementation on cognition is limited. A recent meta-analysis [60], which included randomised or quasi-randomised placebo-controlled trials of vitamin and mineral supplementation for preventing dementia or delaying cognitive decline among patients with mild cognitive impairment, found only one trial, which included combined vitamin E and C supplementation in 256 patients and found no conclusive data for supplementation, reducing the risk of progression to dementia due to very low-quality evidence. Due to this research gap, it will be difficult to determine whether the routine determination of vitamin C status in patients with cognitive impairment, let alone its supplementation, is a useful and cost-effective strategy.

### Limitations

The results of this study should be interpreted with caution because it included only older inpatients receiving rehabilitation, and our findings may not be applicable to a relatively healthy community-dwelling older population. The cross-sectional design of this study does not point towards causality. In addition, we used MMSE to assess cognition, a tool that is regarded as less sensitive for the detection of mild cognitive impairment compared to other tools such as MOCA [54]. We used the total MMSE score to assess cognition. The impact of vitamin C deficiency on specific areas of cognition involved in the performance of the MMSE such as orientation, attention, concentration and short-term memory, was not determined. However, despite the use of a less sensitive tool, our study found an association between vitamin C deficiency and cognitive impairment in a vulnerable cohort of older hospitalised patients.

## 5. Conclusions

Vitamin C deficiency is common, and there are few clinical correlates that can usefully lead to the identification of this condition in older hospitalised patients. Vitamin C deficiency is associated with cognitive impairment, and further studies are needed to confirm and characterise this association in greater detail.

## Figures and Tables

**Table 1:** Characteristics of patients with and without vitamin C deficiency.

Variable	Vitamin C Not Deficient ≥11 μmol/L	Vitamin C Deficient <11 μmol/L	*p*-Value
**N (%)**	118 (73.7)	42 (26.3)	
**Age**	84.4 (6.3)	84.5 (6.6)	0.969
**Sex male**	46 (38.9)	18 (42.8)	0.660
**Income < 40 k/year *n* (%)**	70 (59.8))	24 (57.1)	0.761
**Education university degree *n* (%)**	52 (44.1)	13 (30.9)	0.357
**Married/defacto *n* (%)**	73 (62.4)	23 (54.8)	0.386
**Living status alone *n* (%)**	66 (55.9)	16 (38.1)	0.047
**Current smokers *n* (%)**	3 (2.5)	4 (9.5)	0.041
**Alcohol drinks/week mean (SD)**	1.8 (2.9)	0.9 (2.6)	0.106
**Fruits/vegetable intake/day mean (SD)**	1.3 (0.6)	1.2 (0.5)	0.989
**Charlson index mean (SD)**	8.1 (2.5)	9.2 (2.7)	0.021
**CDT *n* (%)**	76 (64.4)	33 (78.6)	0.091
**Medication number mean (SD)**	7.2 (3.6)	8.4 (3.6)	0.078
**Polypharmacy, *n* (%)**	77 (65.3)	31(73.8)	0.309
Patients prescribed with medications with anticholinergic activity *	62 (52.5)	18 (42.9)	0.281
**Scurvy symptoms *n* (%)**	54 (45.8)	26 (61.9)	0.072
**BMI in kg/m^2^ mean (SD)**	26.2 (5.8)	26.6 (4.7)	0.700
**MUST score mean (SD)**	0.9 (1.2)	0.8 (1.1)	0.404
**MMSE score mean (SD)**	24.9 (3.4)	23.6 (3.3)	0.030
**GDS scores mean (SD)**	4.5 (2.8)	4.5 (2.6)	0.964
**Depression *n* (%)**	43 (36.4)	18 (42.9)	0.462
**EFS score, mean (SD)**	9.6 (2.2)	10.3 (1.9)	0.070
**Frail *n* (%)**	97 (82.2)	38 (90.5)	0.321
**HARP mean (SD)**	3.0 (1.1)	3.1 (0.9)	0.494
**Haemoglobin g/L mean (SD)**	118.0 (16.8)	114.8 (17.4)	0.293
**Albumin g/L mean (SD)**	34.1 (22.9)	30.3 (6.9)	0.295
**Creatinine μmol/L μmol/L**	88.3 (35.5)	110.6 (56.4)	0.003
**Vitamin C μmol/L mean (SD)**	34.3 (22.4)	5.6 (2.4)	<0.001
**Vitamin D nmol/L mean (SD)**	68.9 (31.4)	66.8 (30.8)	0.705
**Vitamin B12 pmol/L mean (SD)**	491.8 (348.5)	436.5 (349.9)	0.355

* Medications with anticholinergic activity such as antihistamines, anti-parkinson, opiates, antimuscarinic, antipsychotic and antiepileptic drugs. SD, standard deviation; CDT, clock drawing test; BMI, body mass index; MUST, malnutrition universal screening tool; MMSE, mini mental state examination; GDS, geriatric depression scale; EFS, Edmonton frail scale; HARP, hospital admission risk profile score.

**Table 2:** Logistic regression model comparing patients with vitamin C deficiency with non-vitamin C deficient patients and cognitive impairment as an outcome variable.

Variable	aOR	95% CI	*p*-Value
**Vitamin C deficiency**	2.93	1.05–8.19	0.031
**Age**	1.02	0.94–1.09	0.658
**Sex male**	0.64	0.22–1.83	0.407
**Living alone**	5.30	1.81–15.19	0.002
**Charlson index**	1.16	0.97–1.39	0.111
**MUST score**	1.15	0.81–1.64	0.444
**Depression**	1.89	0.86–4.14	0.115
**Education university degree**	0.51	0.26–0.99	0.048
**Income < $40,000/year**	2.50	0.93–6.72	0.068
**HARP score**	1.92	1.19–3.09	0.008
**Polypharmacy**	0.53	0.23–1.19	0.123
**Fruit/vegetable intake**	1.02	0.91–1.14	0.707
**Smokers**	1.46	0.65–3.27	0.357
**Haemoglobin**	0.99	0.96–1.01	0.271
**Vitamin D**	0.99	0.99–1.01	0.745
**Vitamin B12**	1.00	0.99–1.00	0.889

aOR, adjusted odds ratio; CI, confidence interval; MUST, malnutrition universal screening tool; HARP, hospital admission risk profile score.

## Data Availability

The data presented in this study are available on request from the corresponding author. The data are not publicly available due to ethical reasons.
